# Supplementary material for: A bio-functional polymer that prevents retinal scarring through modulation of NRF2 signalling pathway
Source: Nat Commun. 2022 May 19;13:2796. doi: 10.1038/s41467-022-30474-6 (PMC9119969; doi:10.1038/s41467-022-30474-6)
Supplement: Supplementary file 1 — Supplementary Information [file 41467_2022_30474_MOESM1_ESM.pdf]

Table of Contents

Supplementary Fig. 1 | Synthesis and characterisation of poly(CEP) using nuclear magnetic resonance (NMR) and gel permeation chromatogram (GPC).
Supplementary Fig. 2 | *In vivo* ophthalmic follow-up of air-filled PVR model and after treatment with poly(CEP) or SF_6 gas.
Supplementary Fig. 3 | Multimodal ophthalmic follow-ups and histology of controls.
Supplementary Fig. 4 | Characterisation of poly(CEP) micelles.
Supplementary Fig. 5 | Micelle shedding from poly(CEP) hydrogel monitored using DPH.
Supplementary Fig. 6 | RPE identity genes (*Rpe65* and *Otx2)* and epithelial junction genes (*Tjp1* and *Cdh1)* were quantified at mRNA level.
Supplementary Fig. 7 | RNA-Seq after 1wt% poly(CEP) exposure.
Supplementary Fig. 8 | Widespread transcriptomic changes mediated by 1wt% poly(CEP) micelles at 8 h.
Supplementary Fig. 9 | Differentially regulated genes in NRF2 pathway at 8 h time point.
Supplementary Fig. 10 | | Differentially regulated genes in NRF2 pathway in retina and RPE-choroid tissues.
Supplementary Fig. 11 | Flow cytometry gating strategy.

Supplementary Table 1 | Calculation of PEG, PPG, PCL, and HMDI compositions in poly(CEP) based on NMR integrals of the respective characteristic peaks.
Supplementary Table 2 | Summary of animal study.
Supplementary Table 3 | Correlation of log2 RNA-Seq fold change (fc) values to log2 RT-qPCR fc values.
Supplementary Table 4 | Primary and secondary antibodies list.
Supplementary Table 5 | Primer list.

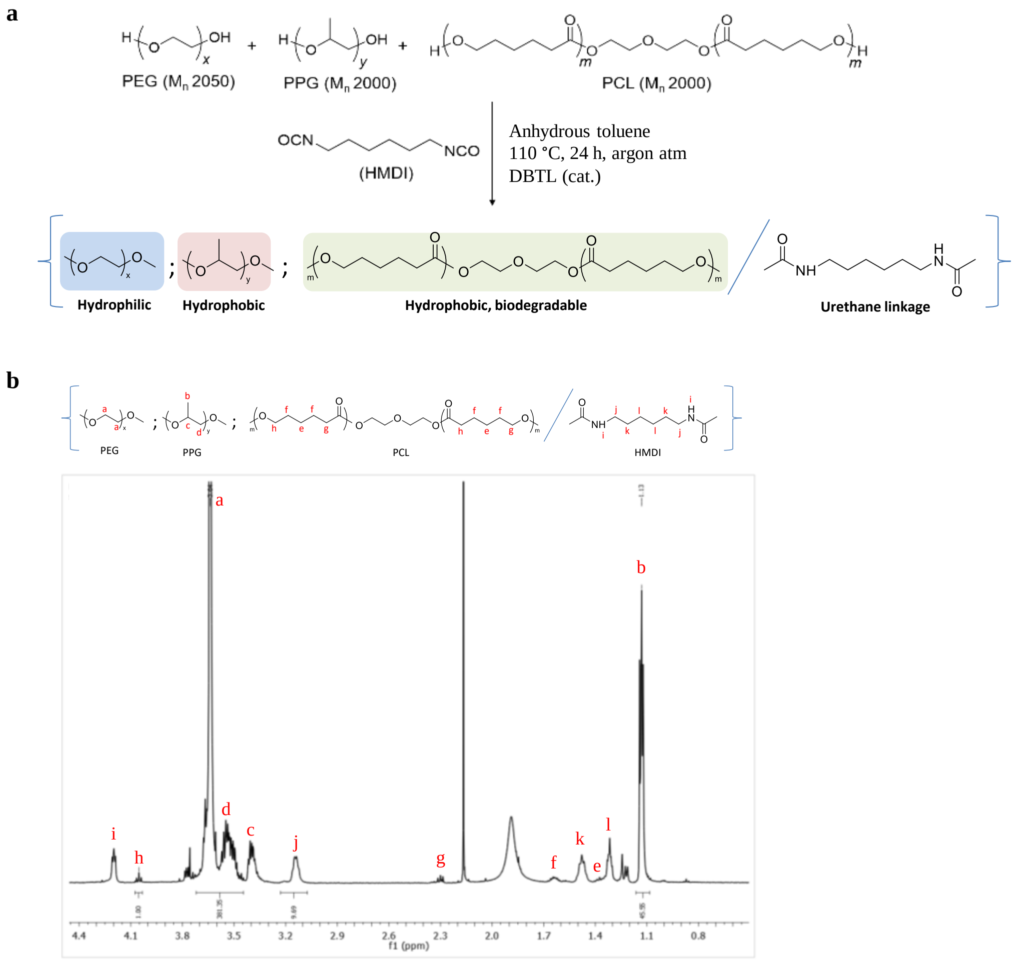

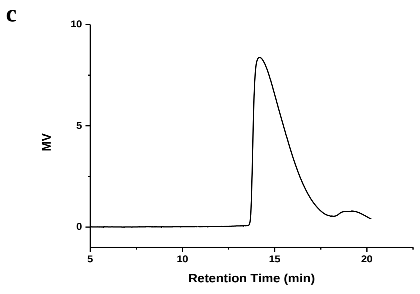

Sample	Mn	Mw	PDI
EPC	26700	38800	1.45

Supplementary Fig. 1 | Synthesis and characterisation of poly(CEP) using nuclear magnetic resonance (NMR) and gel permeation chromatogram (GPC). (**a**) Schematic representation of poly(PEG/PPG/PCL) (termed poly(CEP)) synthesis and chemical structure. (**b**) The corresponding protons from the NMR spectrum were identified and integration ratios of the characteristic PEG, PPG, PCL, and HMDI peaks shown in Table S1. (**c**) GPC was done in tetrahydrofuran (THF) at 40 °C using a Waters GPC system equipped with refractive index detector. The number average (Mn), weight average (Mw), and polydispersity index (PDI) of poly(CEP) was obtained. MV = millivolts.

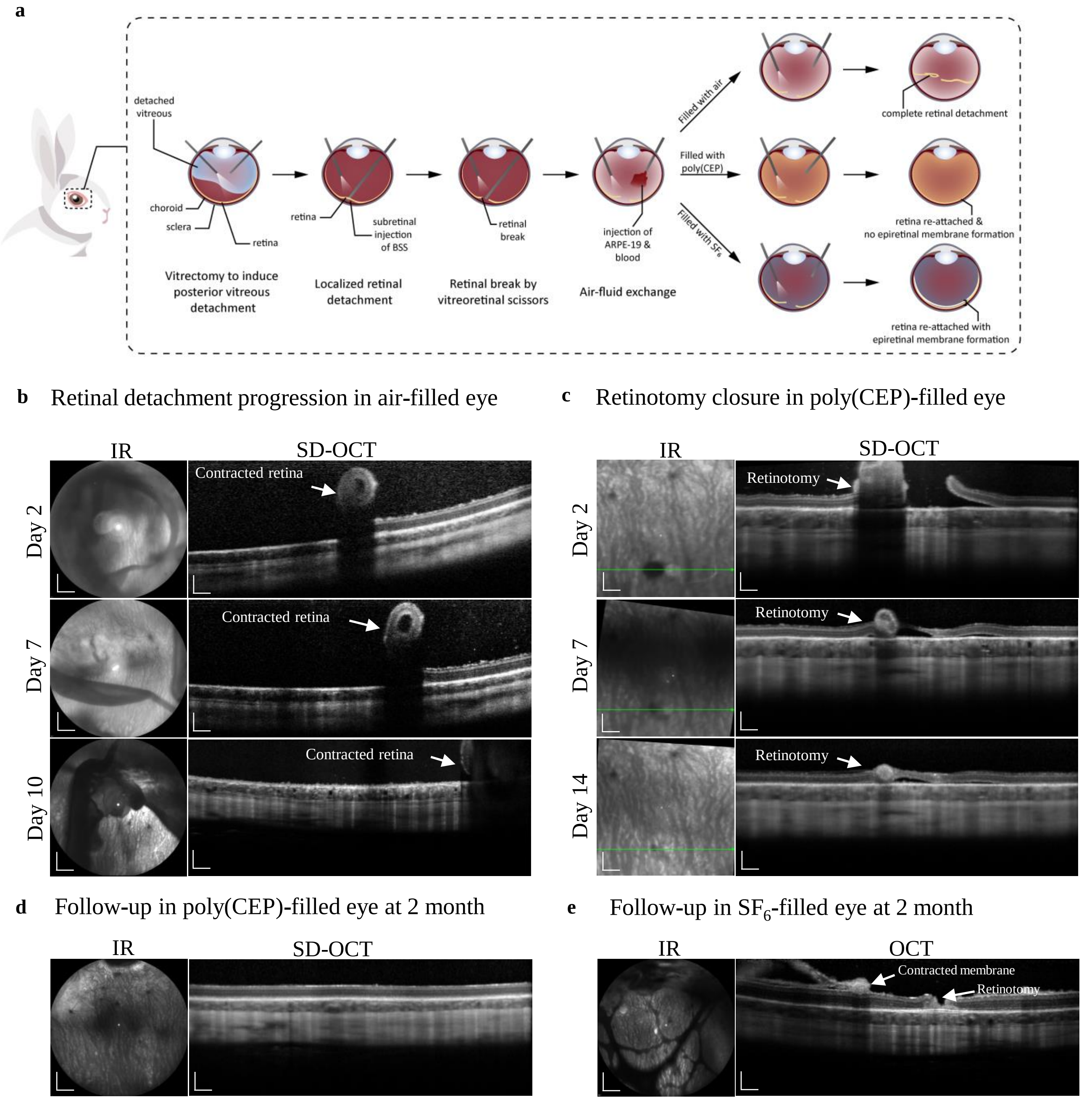

Supplementary Fig. 2 | *In vivo* ophthalmic follow-up of air-filled PVR model and after treatment with poly(CEP) or SF_6 gas. (**a**) Schematic diagram of establishing the rabbit model of PVR and treatment with 10wt% poly(CEP) or 20% SF_6. (**b**) RD in air-filled eye (*n*=5) (experimental PVR model) was followed up to day 10 using IR and SD-OCT showing contracted and detached retina. (**c**) In the poly(CEP)-filled eye (*n*=8), the retinotomy created during the induction of PVR was seen to be partially closed at day 14. (**d**) At 2 months, poly(CEP)-filled eye (*n*=4) showed a clear IR image and a normal retinal structure with healed retinotomy, indicating the protection conferred by poly(CEP) against PVR. (**e**) SF_6-filled eye (*n*=3) showed black shadows in the IR image indicating presence of membranes, which was further confirmed by the SD-OCT image. Retinotomy site is not closed. Scale bar, 2 mm in IR images, 200 μm in OCT images.

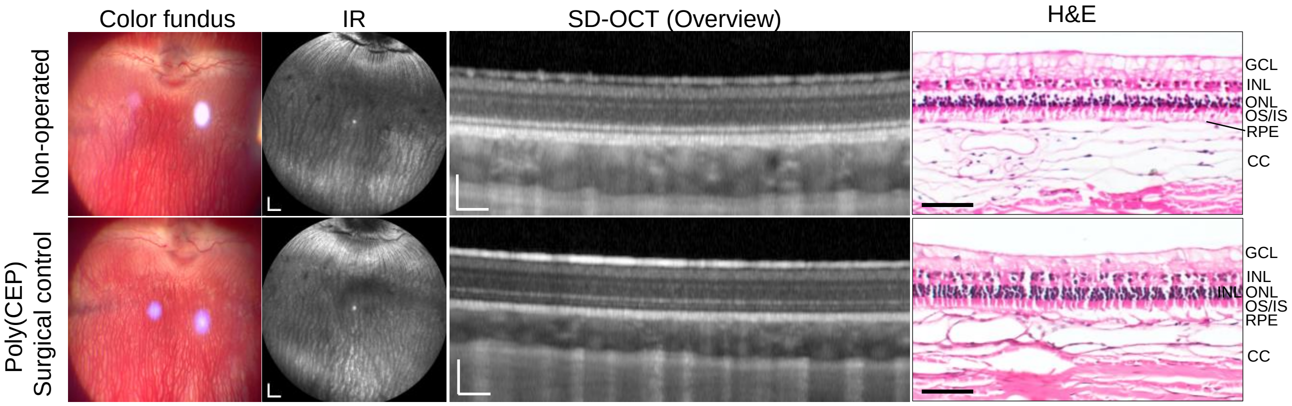

Supplementary Fig. 3 | Multimodal ophthalmic follow-ups and histology of controls. Color fundus and IR images showed poly(CEP) was transparent in vitreous cavity with normal vasculature. Retinal structure and thickness were well-maintained in poly(CEP)-surgical control group (*n*=5) in SD-OCT and H&E images at 2 months post-surgery, and comparable to non-operated control (*n*=5). GCL = ganglion cell layer. INL = inner nuclear layer. ONL = outer nuclear layer. OS/IS = outer segment/ inner segment. RPE = retinal pigment epithelium. CC = Choriocapillaris. Scale bar for IR panel, 2 mm; SD-OCT panel, 200 μm; H&E panel, 100 μm.

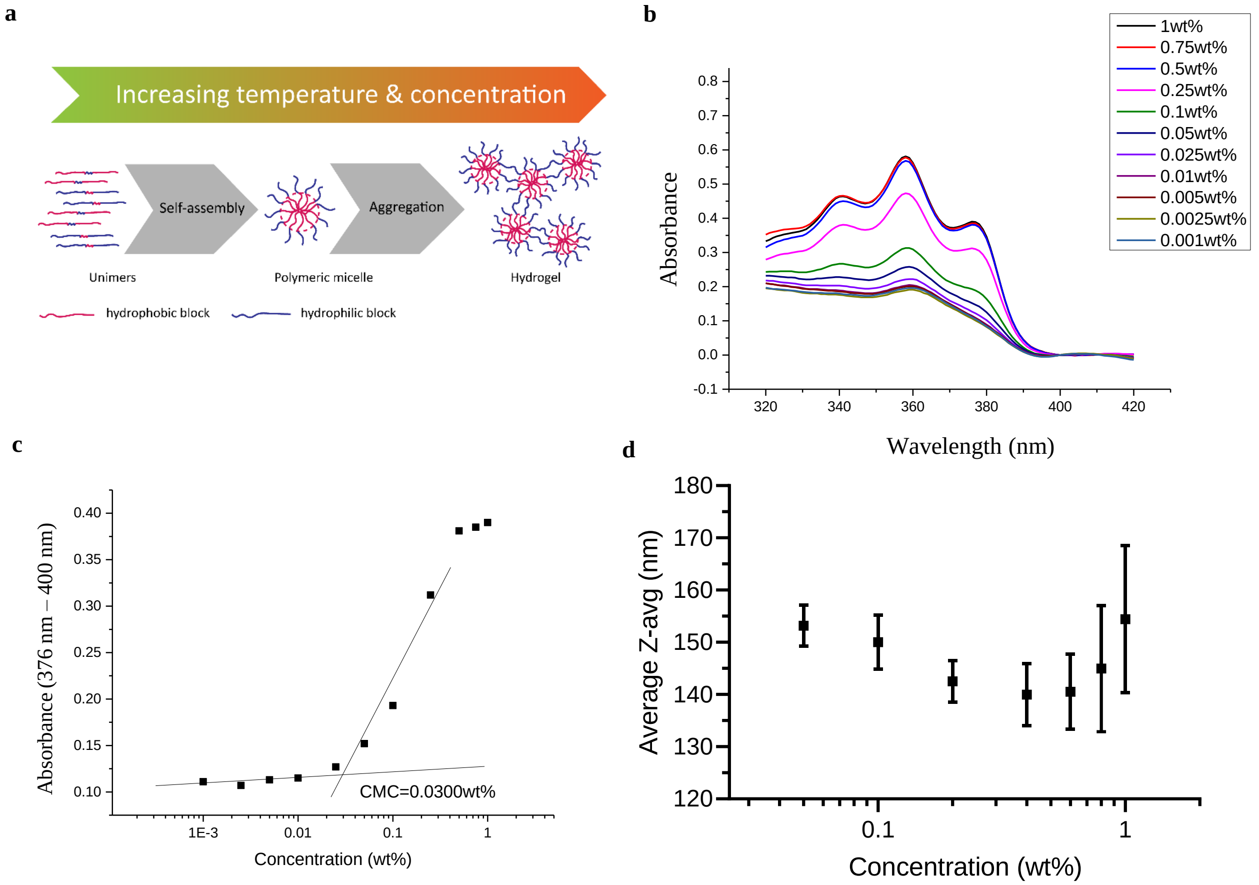

Supplementary Fig. 4 | Characterisation of poly(CEP) micelles. (**a**) Schematic showing three phases of poly(CEP) (unimer, polymeric micelle and hydrogel) dependent on temperature and concentration. (**b**) The absorbance spectra of 1,6-diphenyl-1,3,5-hexatriene (DPH) when added to increasing concentrations of poly(CEP) in basal cell culture medium at 37 °C (body temperature). Characteristic absorbance peaks of DPH are observed at 344, 358 and 376 nm and absorbance increases with higher concentrations. (**c**) Determination of critical micelle concentration (CMC) by extrapolating the difference in DPH absorbance at wavelengths 376 nm and 400 nm in the unimeric and micellar regions. CMC = 0.03 wt%. (**d**) Average sizes (Z-avg) of poly(CEP) micelles at various concentrations measured by dynamic light scattering. Data represents mean ± s.d. of three replicates.

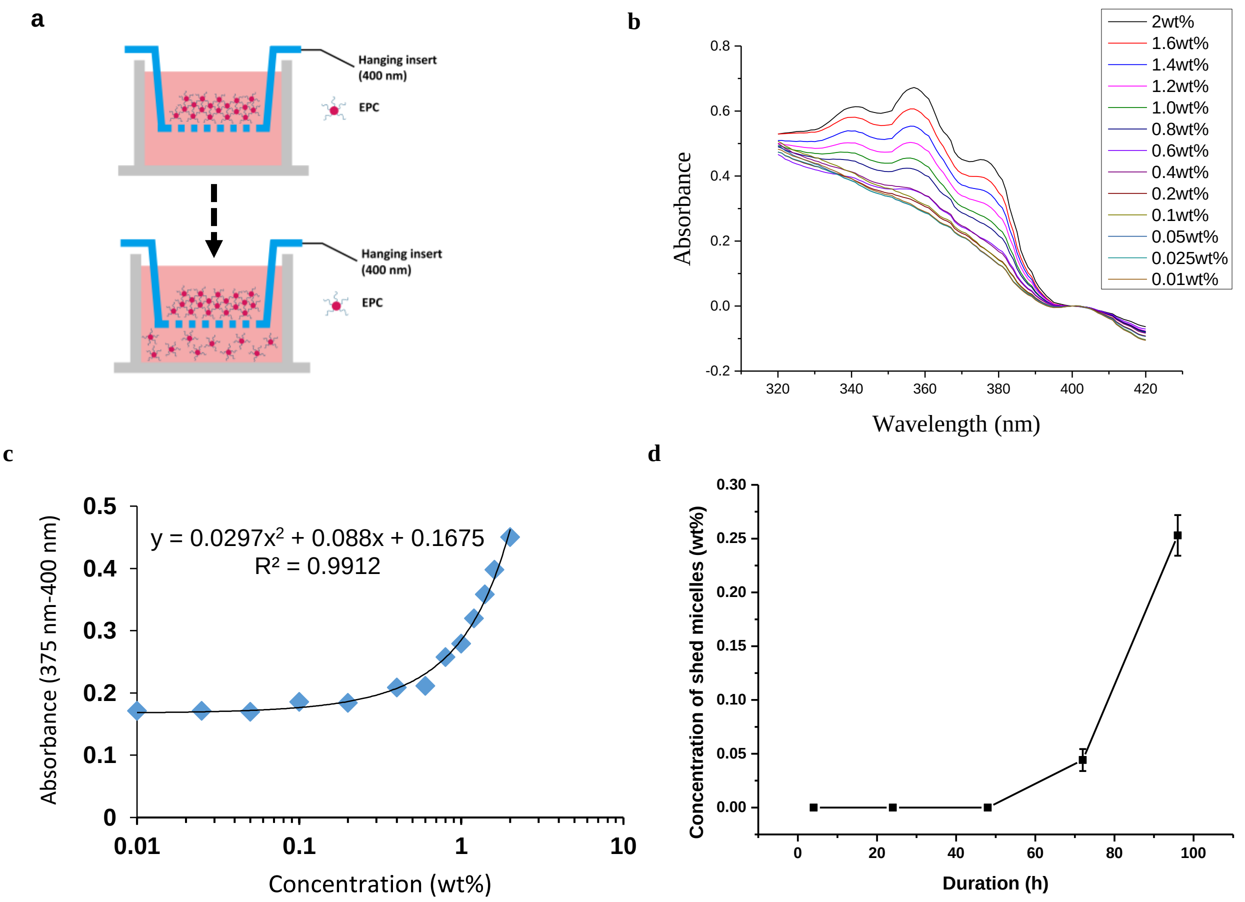

Supplementary Fig. 5 | Micelle shedding from poly(CEP) hydrogel monitored using DPH. (**a**) Schematic of 10wt% poly(CEP) hydrogel placed in hanging cell culture insert and polymeric micelle shedding into the exterior chamber over time. (**b**) DPH absorbance spectra of poly(CEP) micelle solutions at various concentrations in basal media. (**c**) Standard curve of the micelle concentrations against difference in DPH absorbance (375 nm – 400 nm). A quadratic trend line is fitted to the curve; the equation of the trend line is as shown in the graph and the R^2 is 0.9912, suggesting that the trend line fits suitably with the curve. (**d**) Graph showing the concentration of shed micelles (wt%) into the exterior well from 10wt% hydrogel placed in hanging inserts. Significant surface erosion and micelle shedding is observed after 48 h and continued increasing until 96 h. Data represents mean ± s.d. of three replicates.

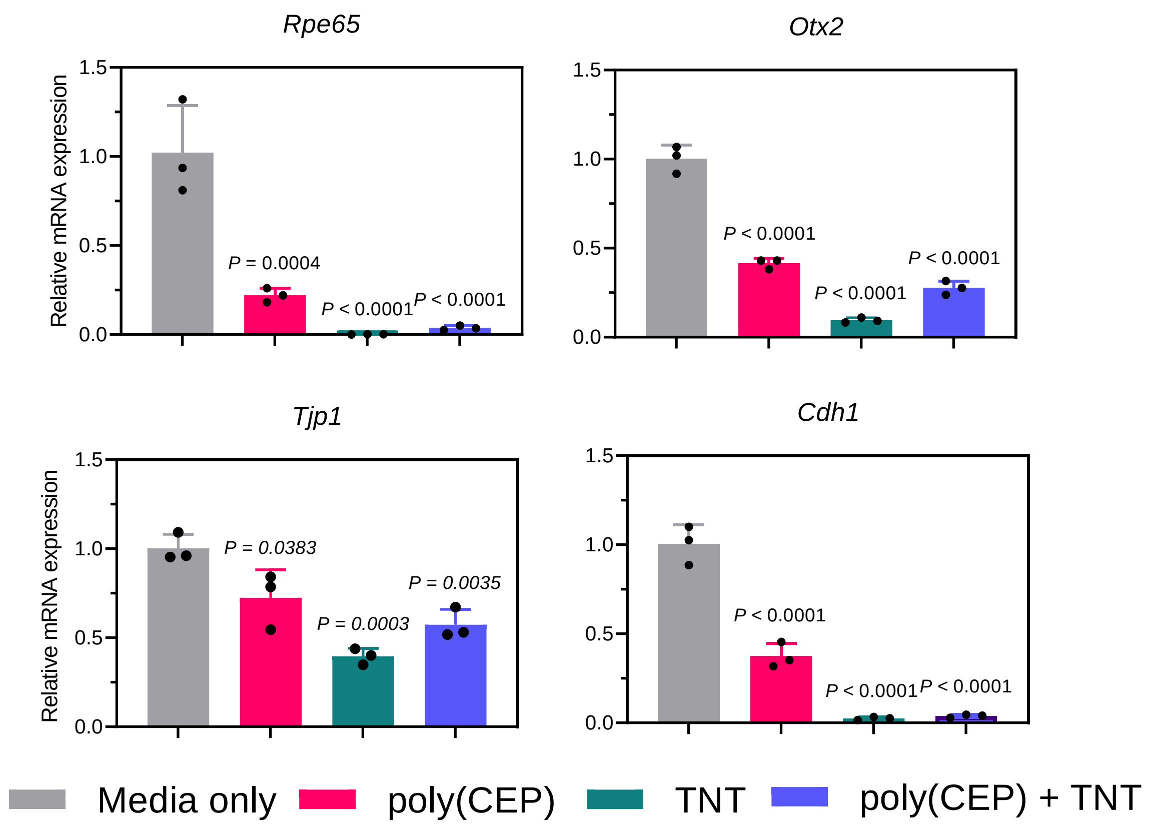

Supplementary Fig. 6 | RPE identity genes (*Rpe65* and *Otx2)* and epithelial junction genes (*Tjp1* and *Cdh1)* were quantified at mRNA level. Reduction of all four genes was observed with the addition of TNT, which was not reversed by poly(CEP) + TNT. Data represents mean ± s.d. of three replicates for RT-qPCR. Statistical analysis was calculated using one-way ANOVA, followed by Tukey's honest significance difference (HSD) *post-hoc* test.

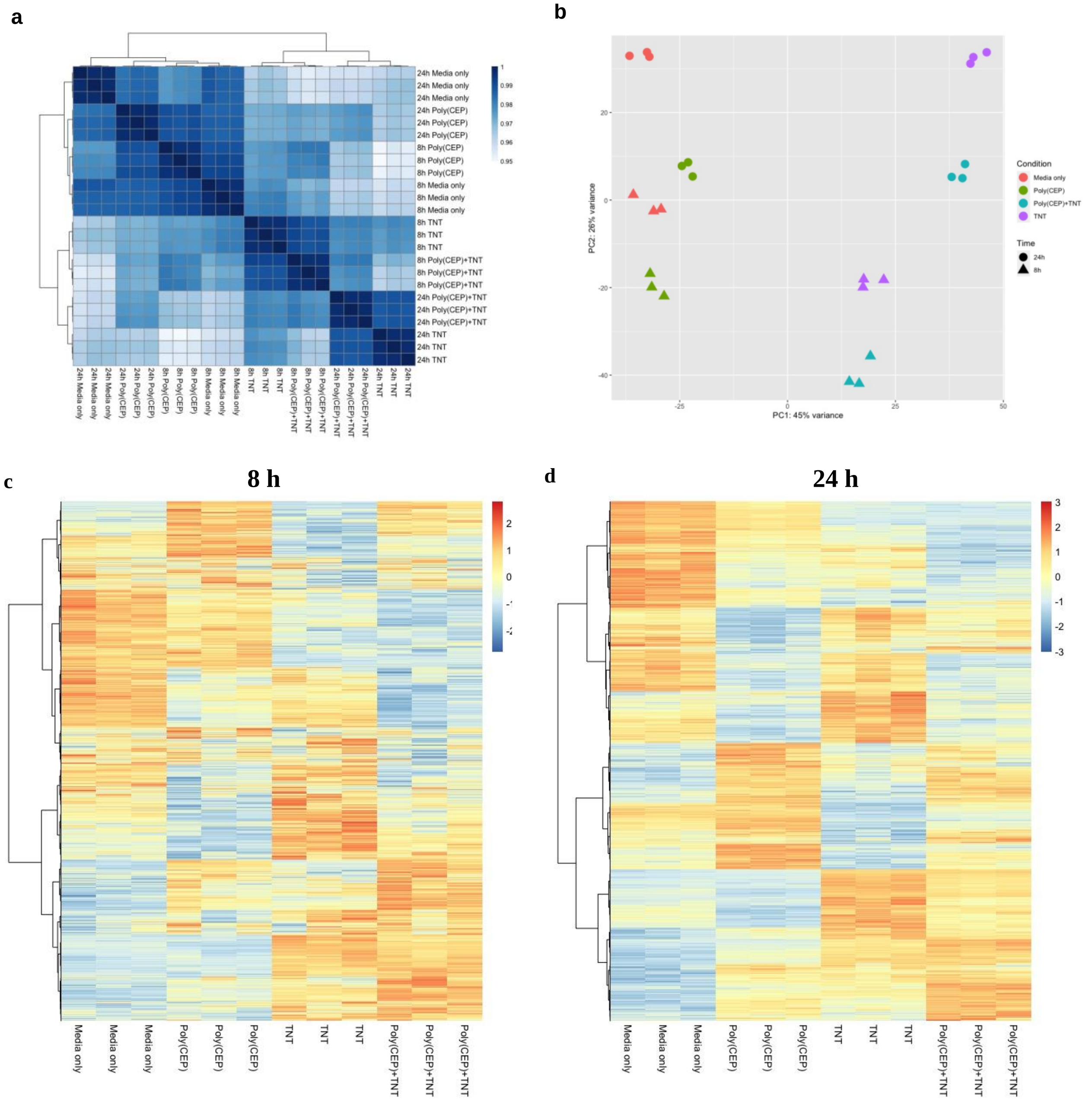

Supplementary Fig. 7 | RNA-Seq after 1wt% poly(CEP) exposure. (**a**) Correlation heatmap of gene expression profiles from RNA sequencing of all four groups (Media only, 1 wt% poly(CEP), TNT and 1 wt% poly(CEP) + TNT at two time points (8 and 24 h), each done in triplicates. The intensity of each box corresponds to the pairwise Pearson's correlation coefficient value shown in the legend. (**b**) Principal component analysis (PCA) of all samples. Triplicates of each condition within same timepoint group together, revealing minimal gene expression variation between triplicates. (**c** and **d**) Hierarchical clustering heatmaps of differentially expressed genes *(*Wald test, Benjamini-Hochberg (BH) $P_{adj} < 0.05$) identified by comparing all four groups at 8 and 24 h.

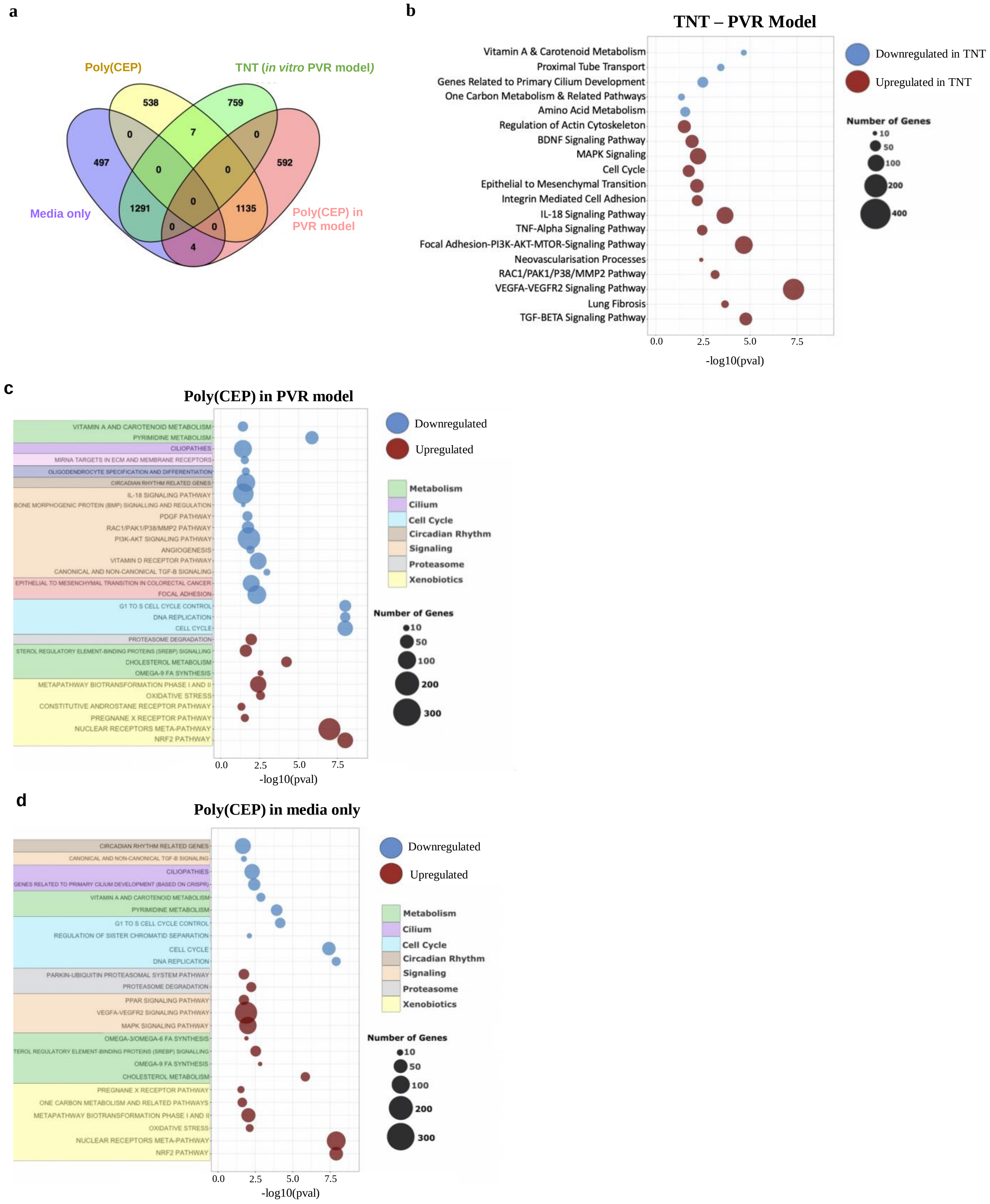

Supplementary Fig. 8 | Widespread transcriptomic changes mediated by 1wt% poly(CEP) micelles at 8 h. (**a**) Venn diagram showing overlap of DEGs (Wald test, BH P_{adj}<0.05) at 8 h identified by DESeq2 at 8 h post-exposure to poly(CEP), TNT or poly(CEP) + TNT. (**b**) GSEA was performed on the *in vitro* PVR model induced by TNT, which showed upregulation of classical pathways involved in PVR pathogenesis. (**c** and **d**) GSEA was used to identify upregulated and downregulated pathways at 8 h after poly(CEP) in PVR model or in media only.

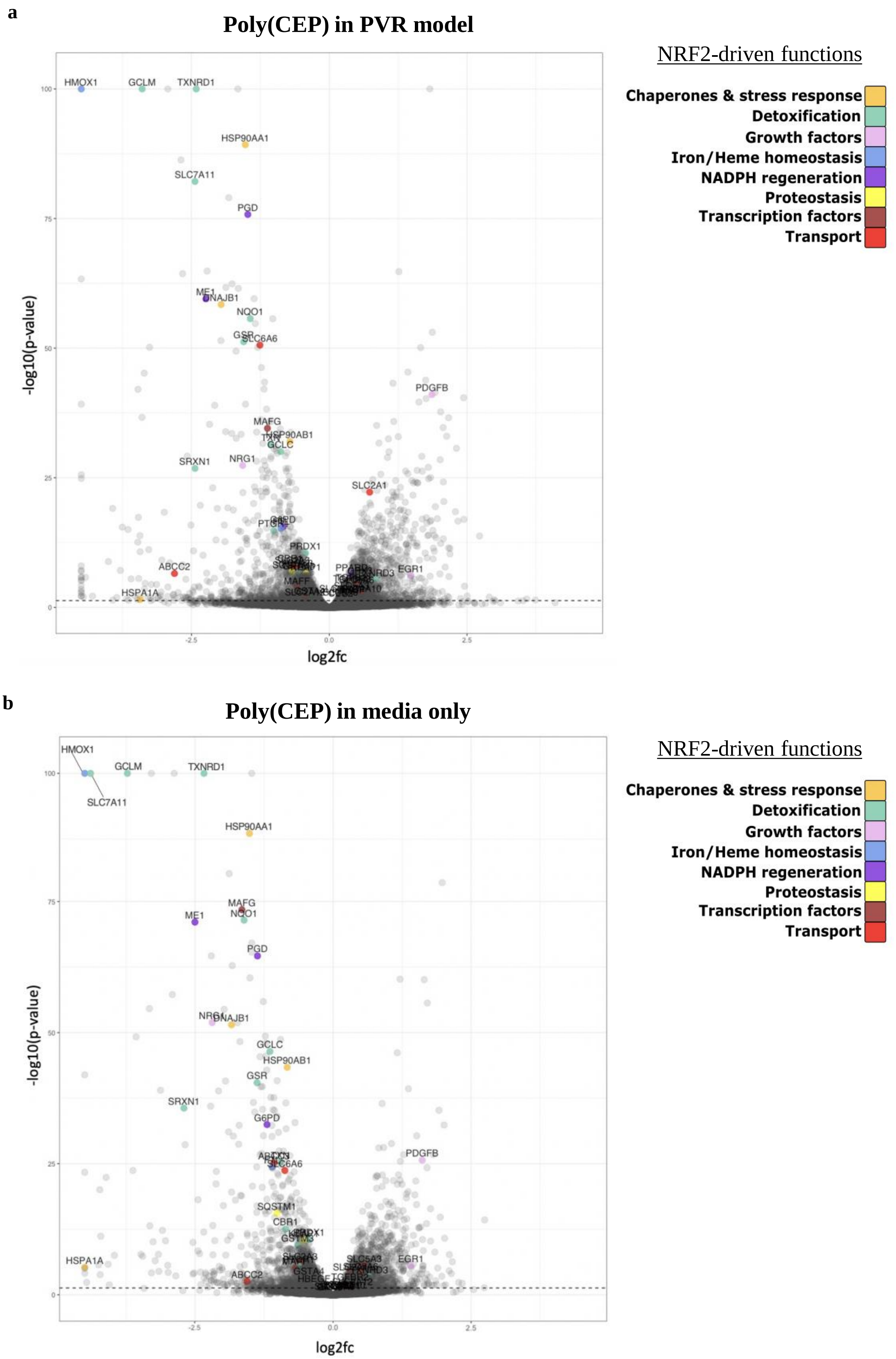

Supplementary Fig. 9 | Differentially regulated genes in NRF2 pathway at 8 h time point. (**a** and **b**) Volcano plot showed >40 genes in NRF2 signaling cascade (highlighted in colour) were upregulated at 8 h after poly(CEP) exposure in the presence and absence of TNT. *P*-values were calculated using the Wald test. The BH method was used to adjust *p*-value for multiple comparisons. Dashed line indicates $P_{adj} = 0.05$.

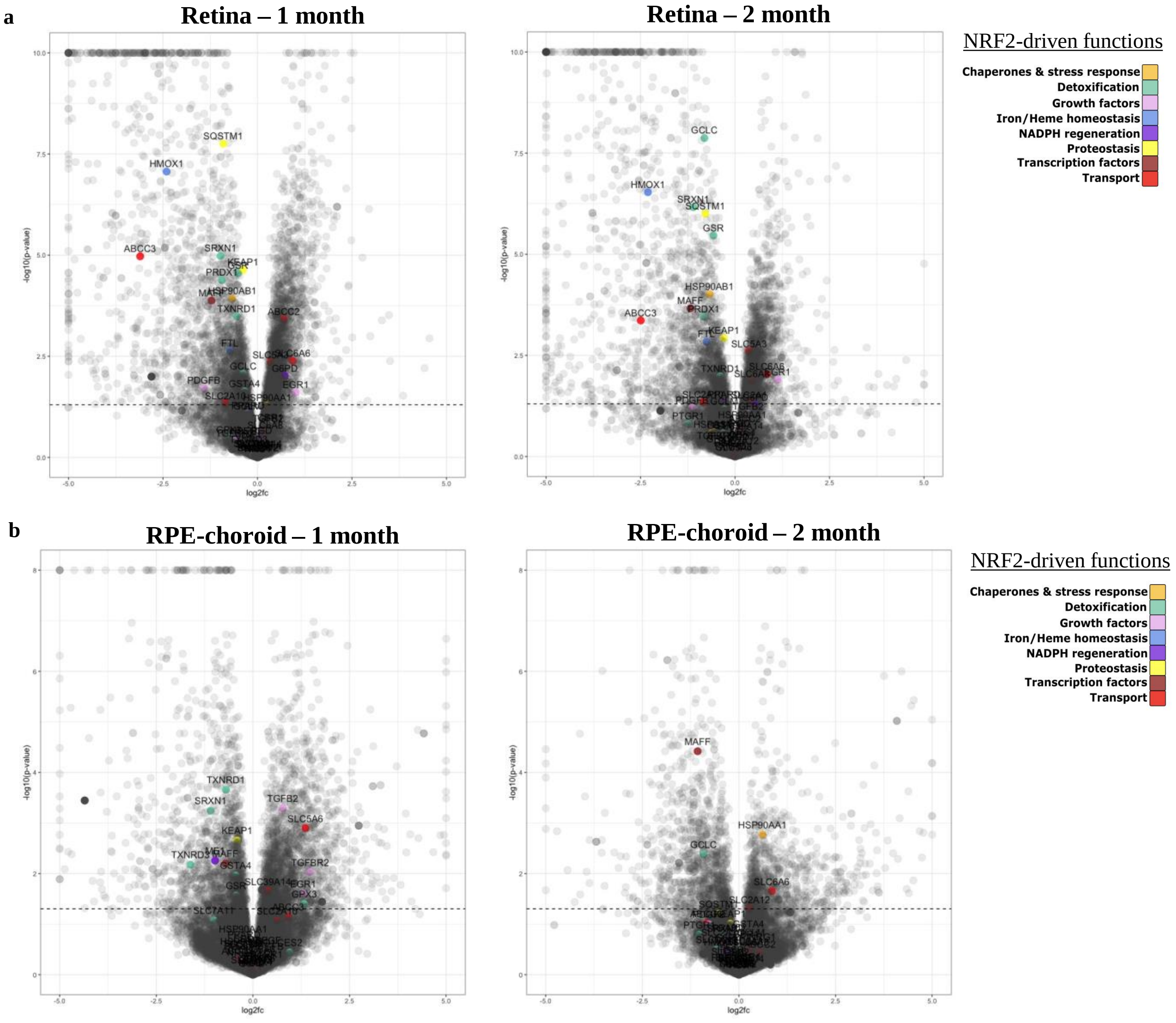

Supplementary Fig. 10 | Differentially regulated genes in NRF2 pathway in retina and RPE-choroid tissues. Volcano plot showed significant upregulation of NRF2 target genes (highlighted in colour) in both retina (**a**) and RPE-choroid (**b)** tissues at 1-month and 2-months after vitreous replacement with poly(CEP). *P*-values were calculated using the Wald test. The BH method was used to adjust *p*-value for multiple comparisons. Dashed line indicates $P_{adj} = 0.05$.

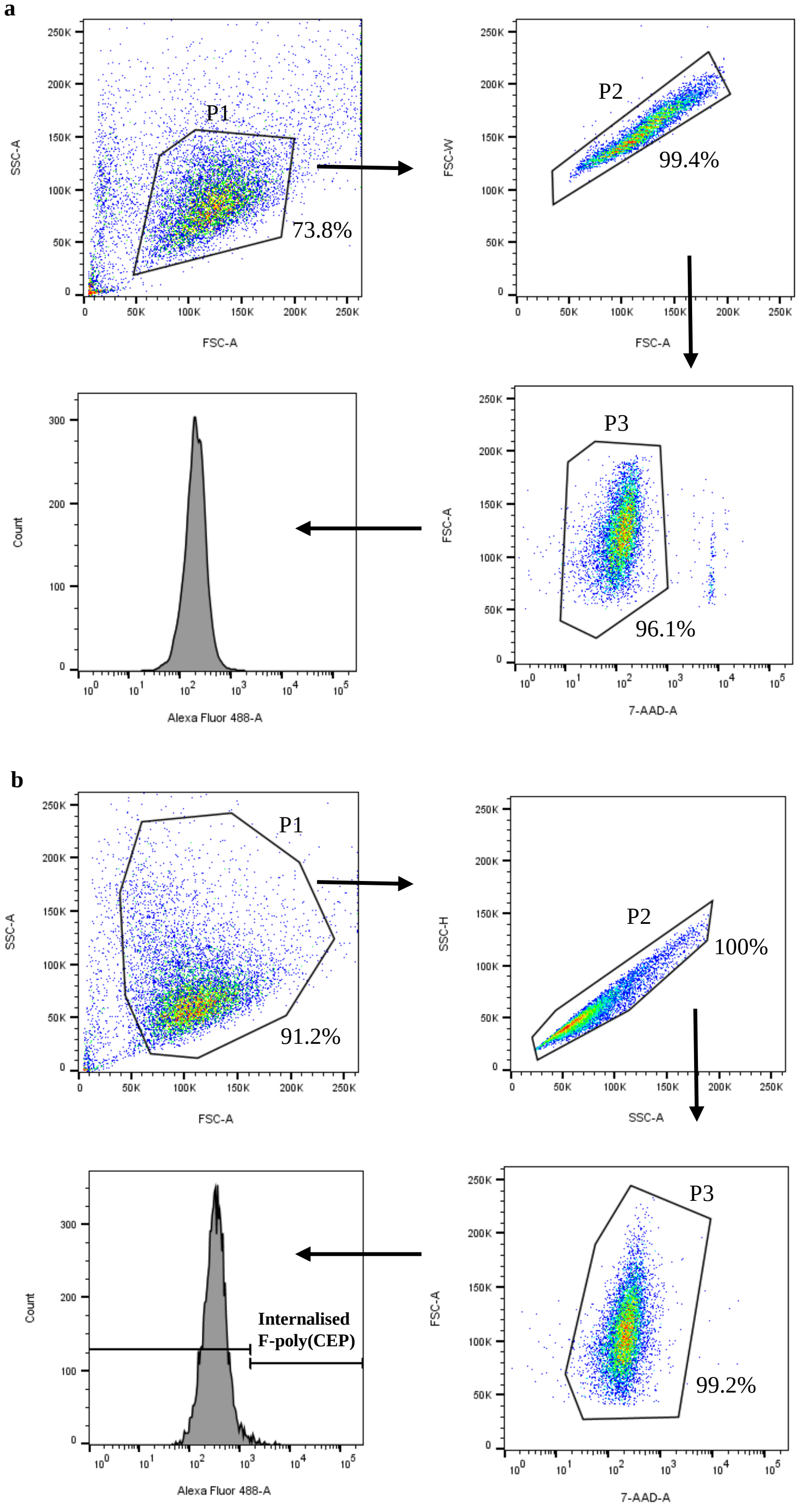

Supplementary Fig. 11 | Flow cytometry gating strategy. Gating was performed by first selecting all cells (P1, exclusion of debris and large clumps) followed by exclusion of doublets (P2), and selection of live cells by exclusion of 7-AAD positive cells (P3) and in (**a**) measurement of MFI (Alexa Fluor 488 channel) or (**b**) gating of Alexa Fluor 488 histogram for % of internalised F-poly(CEP).

Supplementary Table 1 | Calculation of PEG, PPG, PCL, and HMDI compositions in poly(CEP) based on NMR integrals of the respective characteristic peaks. PEG = poly(ethylene glycol). PPG = poly(propylene glycol). PCL = poly(ε-caprolactone). HMDI = hexamethylene diisocyanate. NMR = nuclear magnetic resonance.

**Macromonomer**	**PEG**	**PPG**	**PCL**	**HMDI**
Mass of macromonomer	2050	2000	2000	168
Mass of repeating unit	44	58	114	168
No. of repeating units per macromonomer	46.2	34.2	17.4	1
No. of protons per repeating unit contributing to specified peak	4	3	2	4
No. of protons per macromonomer contributing to specified peak	185	103	34.8	4
Relative NMR integrals	351	45.6	1	9.69
Mole ratio of macromonomers in polymer	1.90	0.44	0.0288	2.42
Normalised mole ratio of macromonomers in polymer	4.27	1	0.0647	5.45

Supplementary Table 2 | Summary of animal study. Rabbit model of PVR was established by creating a local RD and co-injecting ARPE-19 cells and rabbit blood. Rabbit eyes filled with air served as positive control (*n*=5, Case #1-5). Experimental rabbit eyes were then either filled with poly(CEP) (*n*=8, Case #6-13) or SF_6 gas (*n*=5, Case #14-18). Eyes (*n*=8, Case #19-21, #4, #12, #13, #17, #18 contralateral eyes) filled with poly(CEP) after vitrectomy, without PVR model creation (inducing RD, injection of ARPE-19 cell together with rabbit blood), served as an additional surgical control. RD = retinal detachment. ERM = epiretinal membrane.

Case ID	Tamponade	*In vivo* follow-up findings	PVR grading	Follow-up duration
#1	Air	Total RD and contracted retina	5	14 days
#2	Air	Total RD and contracted retina	5	14 days
#3	Air	Total RD and contracted retina	5	2 months
#4	Air	Total RD and contracted retina	5	2 months
#5	Air	Partial RD and contracted retina	5	2 months
#6	Poly(CEP)	Presence of intravitreal membrane, No RD	1	14 days
#7	Poly(CEP)	Presence of intravitreal membrane, No RD	1	14 days
#8	Poly(CEP)	Presence of intravitreal membrane, No RD	1	14 days
#9	Poly(CEP)	Presence of intravitreal membrane, No RD	1	14 days
#10	Poly(CEP)	Presence of ERM but no retinal traction	1	2 months
#11	Poly(CEP)	Presence of ERM with presence of focal retinal traction	2	2 months
#12	Poly(CEP)	Presence of intravitreal membrane, no RD	1	2 months
#13	Poly(CEP)	Presence of intravitreal membrane, no RD	1	2 months
#14	SF_6	Presence of ERM with presence of focal retinal traction .	2	14 days
#15	SF_6	Presence of ERM with presence of focal retinal traction .	2	14 days
#16	SF_6	Presence of ERM with presence of focal retinal traction	2	2 months
#17	SF_6	Presence of ERM with presence of focal retinal traction	2	2 months
#18	SF_6	Total RD and contracted retina	5	2 months
#19	Poly(CEP) Without PVR induction	Clear fundus view, normal retinal structure	0	1 month
#20	Poly(CEP) Without PVR induction	Clear fundus view, normal retinal structure	0	1 month
#21	Poly(CEP) Without PVR induction	Clear fundus view, normal retinal structure	0	1 month
#4 Contralateral eye	Poly(CEP) Without PVR induction	Clear fundus view, normal retinal structure	0	2 months
#12 Contralateral eye	Poly(CEP) Without PVR induction	Clear fundus view, normal retinal structure	0	2 months
#13 Contralateral eye	Poly(CEP) Without PVR induction	Clear fundus view, normal retinal structure	0	2 months
#17 Contralateral eye	Poly(CEP) Without PVR induction	Clear fundus view, normal retinal structure	0	2 months
#18 Contralateral eye	Poly(CEP) Without PVR induction	Clear fundus view, normal retinal structure	0	2 months

Supplementary Table 3 | Correlation of log2 RNA-Seq fold change (fc) values to log2 RT-qPCR fc values.

**Poly(CEP) + TNT** versus **TNT (24 h)**			**Poly(CEP)** versus **Media only (24 h)**		
**Genes**	**log2 RNA-Seq fc**	**log2 RT-qPCR fc**	**Genes**	**log2 RNA-Seq fc**	**log2 RT-qPCR fc**
Nqo1	3.19	4.09	*Nqo1*	3.12	3.21
Ptgr1	1.53	1.74	*Ptgr1*	1.47	0.98
Gstm3	1.07	0.39	*Gstm3*	0.85	0.46
Abcc3	2.71	2.11	*Abcc3*	4.28	1.99
Slc7a11	4.12	4.28	*Slc7a11*	4.95	3.99
Gclm	2.45	2.75	*Gclm*	3.2	2.3
Gsr	1.2	1.48	*Gsr*	1.58	0.83
Srnx1	0.99	1.53	*Srnx1*	2.99	2.1
Txnrd1	2.33	2.63	*Txnrd1*	2.39	2.36
G6pd	2.24	2.72	*G6pd*	2.93	2.59
Me1	2.02	1.8	*Me1*	2.6	1.69
Hmox1	5.83	5.96	*Hmox1*	6.14	4.99
Ftl	2.33	2.63	*Ftl*	2.67	2.12
Mafg	0.3	0.97	*Mafg*	1.35	0.8
Maff	0.3	0.81	*Maff*	1.35	0.77
Sqstm1	1.37	2.46	*Sqstm1*	1.16	1.56

Supplementary Table 4 | Primary and secondary antibodies list.

Primary antibodies	Supplier	Dilution	Catalogue Number	RRID
Mouse anti-α-SMA	Sigma-Aldrich	1:500	A5228	AB_262054
Mouse anti-CDH-1/ E-Cadherin	BD Biosciences	1:200	610182	AB_397581
Rabbit anti-COL1A1	Sigma-Aldrich	1:500	HPA011795	AB_1847088
Mouse anti-FN1/Fibronectin	Sigma-Aldrich	1:800	F7387	AB_476988
Rabbit anti-Ki67	Abcam	1:1000	Ab15580	AB_443209
Rabbit anti-OTX2	Abcam	1:100	ab92326	AB_10562130
Goat anti-SNAI1/hSnail	R&D Systems	1:200	AF3639	AB_2191738
Rabbit anti-TJP-1/ ZO-1	Invitrogen	1:200	617300	AB_2533938
Rabbit anti-Nrf2	Abcam	1:1000	ab137550	AB_2687540
**Secondary antibodies**	**Supplier**	**Dilution**	**Catalogue Number**	**RRID**
Alexa Fluor 488 Donkey anti-mouse IgG	Invitrogen	1:1000	A21202	AB_141607
Alexa Fluor 488 Goat anti-rabbit IgG	Invitrogen	1:1000	A11008	AB_143165
Alexa Fluor 488 Goat anti-mouse IgG	Invitrogen	1:1000	A11001	AB_2534069
Alexa Fluor 488 Donkey anti-goat IgG	Invitrogen	1:1000	A11055	AB_2534102
Alexa Fluor 568 Donkey anti-rabbit IgG	Invitrogen	1:1000	A10042	AB_2534017

Supplementary Table 5 | Primer list.

Gene	Forward Primer Sequence (5'-3')	Reverse Primer Sequence (5'-3')
Abcc3	CTCCCGATTCCCAACTGAGT	ATCAGAGGGAGGACAGAGGT
Cdh1	ATTTTTCCCTCGACACCCGAT	TCCCAGGCGTAGACCAAGA
Cdh2	AGAGGCTGTCCTTCATGCAC	AGGCGCTGGGGAAAGTATTC
Col1a1	CCCCTGGAAAGAATGGAGATG	TCCAAACCACTGAAACCTCTG
Fn1	GATGCAGACACAGAGCCAAA	CAGTCCTCAGTGGCAGATCA
Foxs1	AGTGGCATCTACCGCTACATC	CACCTTGACAAAGCACTCGT
Ftl	CAGATCCGGGGACTCTCTTC	GGTTGGTTGGCAAGAAGGAG
G6pd	GCATGTTCTTCAACCCCGAG	TCAGGGAGCTTCACGTTCTT
Gapdh	AGCAAGAGCACAAGAGGAAGAG	GAGCACAGGGTACTTTATTGATGG
Gclm	TTCCCATACCACAGCCAAGC	AGAAAGACAGTGTTAAAGGAGCA
Gsr	TGAGTCCTGATCATGCTGCT	GGTTCCCAGCCTCCTCAAAT
Gstm3	GGACCAGAAGCCAAGGATCT	GGCCTGAACACCTAATCCCT
Hmox1	CCATGAACTTTGTCCGGTGG	TGGATGTGCTTTTCGTTGGG
Maff	GACCCCTGCCTTTGATTGTC	CATACGCCACTTTTCCCAGG
Mafg	TGGGTCCAAGCAGAGTTGAT	TTCTCCCACACACACAACCT
Me1	TCCCACCTGAAATCACCTTTT	GCCACAGTCCACAAAATCCC
Nqo1	AGACAGCCTCTTACTTGCCA	AAAACCACCAGTGCCAGTCA
Otx2	AGTTCCGAGAGGCATAGAAGG	TAAGCAGATTGGTTTGTCCAT
Ptgr1	ATAACAGAACCGGCCCACTT	TCAGCAAGTCCTTCAGAGCT
Rpe65	CCTGATTCATACCCATCAGAACCC	CACCACACTCAGAACTACACCATC
Slc7a11	CCAGAATTTCAGGGGCATCG	TGCTTGAGTTGAGGACCAGT
Snai1	ACAAGCACCAAGAGTCCG	ATGGCAGTGAGAAGGATGTG
Snai2	ACTGCTCCAAAACCTTCTCC	TGTCATTTGGCTTCGGAGTG
Srxn1	CATGCATTCTCTTTCCCCGG	GCACTGAAGACACTCTCCCT
Tjp1	GCAGCAATAAAGCAGCGTTTC	TTAGGGCACAGCATTGTATCAC
Txnrd1	GCCTCACGTCCTCATCTCAT	TGGTCAACTGCCTCAATTGC
