# Supplementary material for: A bio-functional polymer that prevents retinal scarring through modulation of NRF2 signalling pathway
Source: Nat Commun. 2022 May 19;13:2796. doi: 10.1038/s41467-022-30474-6 (PMC9119969; doi:10.1038/s41467-022-30474-6)
Supplement: Supplementary file 3 — Description of additional Supplementary File [file 41467_2022_30474_MOESM3_ESM.pdf]

Description of Additional Supplementary Files

File Name: Supplementary Movie 1.

Description: Surgical induction of PVR in a rabbit model by localised retinal detachment, a retinotomy, and co-injection of ARPE-19 cells and blood, followed by treatment with poly(CEP).

File Name: Supplementary Movie 2.

Description: Time-lapse of ES-RPE cells treated with TNF-α and TGF-β (TNT) showing the four stages of RPE transformation over 72 h, ending with contracting membranes.

File Name: Supplementary Movie 3.

Description: Time-lapse of ES-RPE cells treated with 1 wt% poly(CEP) + TNT showing the suppressive effect on RPE transformation over 72 h, and complete prevention of contracting membranes.

File Name: Supplementary Data 1.

Description: Complete DE gene list of TNT versus Poly(CEP) + TNT and Media only versus Poly(CEP) at 8 and 24 h.

File Name: Supplementary Data 2.

Description: GSEA of Media only versus TNT at 8 and 24 h.

File Name: Supplementary Data 3.

Description: GSEA of TNT versus Poly(CEP) + TNT and Media only versus Poly(CEP) at 8 and 24 h.
